# Dipstick proteinuria and risk of myocardial infarction and all-cause mortality in diabetes or pre-diabetes: a population-based cohort study

**DOI:** 10.1038/s41598-017-12057-4

**Published:** 2017-09-20

**Authors:** Jinwei Wang, Junjuan Li, Anxin Wang, Jianli Wang, Yaozheng Yang, Shuohua Chen, Shouling Wu, Minghui Zhao, Xiuhua Guo, Luxia Zhang

**Affiliations:** 1 Renal Division, Department of Medicine, Peking University First Hospital; Institute of Nephrology, Peking University; Key Laboratory of Renal Disease, National Health and Family Planning Commission, Beijing, 100034 China; 2 Key Laboratory of Chronic Kidney Disease Prevention and Treatment, Ministry of Education, Beijing, 100034 China; 3 Department of Nephrology, Kailuan General Hospital Affiliated to North China University of science and technology, Tangshan, 063000 China; 4 Department of Epidemiology and Health Statistics, School of Public Health, Capital Medical University; Municipal Key Laboratory of Clinical Epidemiology, Beijing, 100069 China; 5 Department of Neurology, Beijing Tiantan Hospital, Capital Medical University, Beijing, 100050 China; 6 Department of Cardiology, Kailuan General Hospital Affiliated to North China University of science and technology, Tangshan, 063000 China

## Abstract

To evaluate the association between dipstick proteinuria and myocardial infarction (MI) or all-cause mortality, a cohort study was conducted among 16,573 general Chinese population with diabetes or pre-diabetes, which were defined as self-reported history of diabetes or fasting blood glucose ≥5.6 mmol/L or under blood glucose lowering therapy. Proteinuria was detected biennially during 2006–2013 by dipstick test. MI and all-cause mortality were recorded through the end of 2014. Mean age (standard deviation) of study participants was 51.16 (10.63) years, with 82.24% of male. During a median follow-up of 8.03 years, 211 MI and 403 all-cause mortality occurred. Multivariable Cox regression revealed occasional or persistent detection of trace or higher in proteinuria increased the risk of all-cause mortality, with hazard ratios (HRs) of 1.42 (95% confidence intervals [CI]: 1.10, 1.83) and 2.23 (95% CI: 1.66, 3.01), respectively, compared to sustained negative in proteinuria. A time-dependent analysis also revealed the association between degree of proteinuria and all-cause mortality, with HRs of 1.80 (95% CI: 1.31, 2.48) for trace and 3.34 (95% CI: 2.40, 4.65) for one plus or higher in proteinuria, compared to negative. The associations regarding MI lost statistical significance after multivariable adjustment. In conclusion, dipstick proteinuria was associated with an increased risk of MI and all-cause mortality among a general population with diabetes or pre-diabetes.

## Introduction

Chronic kidney disease (CKD) is a global public health problem. A recent meta-analysis reported the global prevalence of CKD to be 13.4%^1^. At the same time, awareness of CKD is limited worldwide, with nearly 90% of CKD patients unaware of their condition^2–4^. Thus, a simple method for testing CKD indicators may improve the low awareness. Several large-scale investigations have demonstrated that reduced kidney function or increased urine albumin excretion can increase the risk of cardiovascular diseases (CVD), premature death or progression to end stage kidney disease (ESKD). The relationships between the CKD indicators and the adverse outcomes were consistent among general population, high risk population (e.g. population with hypertension and/or diabetes) and CKD patients^5–7^. Hence, it will be reasonable to screen kidney function and/or kidney damage markers among general or specific high risk populations.

Unlike developed countries, most developing countries have their majority of CKD in early stages, typically with normal or mildly reduced kidney function but obvious albuminuria. For example, a nation-wide survey showed China had a prevalence of CKD of 10.8%, meanwhile, the prevalence of albuminuria was as high as 9.4%^8^. Thus, screening for albuminuria may possess more public health meanings. Although albuminuria can be measured by 24h urine protein excretion rate or urine albumin creatinine ratio (ACR), these quantitative methods may not be applicable in areas where health resources are limited. Compared to the above mentioned methods, dipstick test of proteinuria is simpler and more cost-effective. The method was reported to have a good correlation with ACR^9^. A lot of studies have demonstrated that dipstick proteinuria had an equivalent effect on the risk of the adverse outcomes as did ACR^3,5^. Among general populations, there is a study evaluated relation between dipstick proteinuria and risk of myocardial infarction (MI) and all-cause mortality among a province-wide Canadian population^10^, however, evidence from Asians is lacking. There are also further needs to clarify the effect of dipstick proteinuria among specific high risk populations, e.g. people with impaired glucose metabolism.

In the present study, we aimed to investigate the association of dipstick detected proteinuria with risk of MI and all-cause mortality among a large cohort of general Chinese population with diabetes or pre-diabetes. Both the persistence and degree of proteinuria will be evaluated.

## Results

### General characteristics of the population by persistent pattern of proteinuria

Compared to participants excluded from the analysis (n = 13,442), participants included (n = 16,573) were younger, less likely to be male, less likely to be under current anti-hypertensive medication or anti-diabetic medication, more likely to be under higher education level, had higher body mass index (BMI), triglyceride (TG) and estimated glomerular filtration rate (eGFR), lower systolic blood pressure (SBP), diastolic blood pressure (DBP), fasting blood glucose (FBG) and low density lipoprotein cholesterol (LDL-C) (Supplementary Table S1). The mean (standard deviation [SD]) age of the included participants was 51.16 (10.63) years, with 82.24% of male. There are 11,314 (68.3%) participants with pre-diabetes and 5,259 (31.7%) with diabetes. Participants were divided into three categories according to the persistent pattern of positive finding (trace or higher) in proteinuria during follow-up, 12,985 participants with negative proteinuria, 2,636 with occasional positive finding (once during follow-up) and 952 with persistent positive finding (twice or more during follow-up). People detected with occasional or persistent positive finding in proteinuria were older, more likely to be males, under current smoking, under anti-hypertensive treatment, had larger BMI, higher SBP, DBP, FBG, TG, serum creatinine and lower eGFR, compared to people with negative proteinuria (all p-values < 0.05) (Table 1).

**Table 1:** Characteristics of participants stratified by persistent pattern of positive finding in proteinuria.

Characteristics	Total, n = 16573	Persistent pattern of proteinuria during follow-up			p-value
		Negative through follow-up, n = 12985	Occasional trace or higher for once, n = 2636	Persistent trace or higher for two or more times, n = 952	
Age(years)	51.16 (10.63)	50.94 (10.55)	51.34 (10.92)	53.64 (10.68)	<0.001
Male	13629 (82.24%)	10602 (81.65%)	2226 (84.45%)	801 (84.14%)	<0.001
High school or above^*^	3222 (20.01%)	2555 (20.24%)	477 (18.64%)	190 (20.74%)	0.16
Current smoking^*^	5884 (36.24%)	4496 (35.28%)	1025 (39.93%)	363 (39.16%)	<0.001
BMI(kg/m^2^)	25.87 (3.38)	25.72 (3.32)	26.17 (3.48)	26.94 (3.59)	<0.001
SBP(mmHg)	133.82 (20.54)	132.49 (19.88)	136.62 (21.77)	144.16 (22.07)	<0.001
DBP(mmHg)	85.13 (11.62)	84.50 (11.28)	86.60 (12.32)	89.61 (12.69)	<0.001
FBG(mmol/L)^*^	6.96 (2.07)	6.82 (1.90)	7.23 (2.28)	8.17 (3.03)	<0.001
Current use of blood pressure lowering agents^*^	2029 (12.91%)	1377 (11.14%)	411 (16.55%)	241 (27.45%)	<0.001
Current use of glucose lowering agents^*^	1191 (7.51%)	808 (6.49%)	237 (9.42%)	146 (16.39%)	<0.001
TG(mmol/L)	1.48 (1.03, 2.30)	1.44 (1.01, 2.22)	1.56 (1.08, 2.54)	1.89 (1.25, 3.02)	<0.001
LDL-C(mmol/L)^*^	2.44 (0.87)	2.44 (0.85)	2.44 (0.94)	2.38 (1.01)	0.18
Serum creatinine(mg/dl)	1.01 (0.25)	1.01 (0.25)	1.01 (0.26)	1.04 (0.26)	0.002
eGFR(ml/min/1.73 m^2^)	82.26 (68.68, 98.14)	82.43 (68.90, 98.19)	82.86 (69.10, 99.67)	78.75 (65.28, 94.32)	<0.001
eGFR categories					<0.001
≥90 ml/min/1.73 m^2^	6210 (37.52%)	4868 (37.54%)	1031 (39.16%)	311 (32.74%)	
60–90 ml/min/1.73 m^2^	8431 (50.95%)	6652 (51.30%)	1288 (48.92%)	491 (51.68%)	
<60 ml/min/1.73 m^2^	1908 (11.53%)	1446 (11.15%)	314 (11.93%)	148 (15.58%)	

*Note*: Data were presented as mean (standard deviation) or median(interquartile range) for continuous variables and frequency(proportion) for categorical variables.

^*^There are 475 missing value for variable of high school or above, 336 for current smoking, 80 for BMI, 54 for SBP, 54 for DBP, 3 for FBG, 853 ^*^for current use of blood pressure lowering agents, 714 for current use of glucose lowering agents, 31 for TG, 40 for LDL-C and 24 for serum creatinine.

Abbreviations: BMI, Body mass index; SBP, Systolic blood pressure; DBP, Diastolic blood pressure; FBG, Fasting blood glucose; TG, Triglyceride; LDL-C, Low density lipoprotein cholesterol; eGFR, estimated Glomerular filtration rate.

### Incidence rate of MI or all-cause mortality

During a median follow-up of 8.03 years (whole range: 0.05–8.65 years), 211 MI and 403 all-cause mortality events were recorded, with the incidence rates of 1.61 and 3.05 events per 1000 person-years, respectively. The incidence rates of MI and all-cause mortality were both higher among people detected with occasional or persistent positive finding in proteinuria than those with negative proteinuria (all p-values of log-rank test < 0.05) (Table 2).

**Table 2:** Incidence rates for myocardial infarction and mortality among total population and by persistent pattern of positive finding in proteinuria.

Populations	Myocardial infarction		Death	
	Number of Events	Events/1000 person-yrs	Number of Events	Events/1000 person-yrs
Total, n = 16573	211	1.61	403	3.05
Negative through follow-up, n = 12985	142	1.38	259	2.50
Occasional trace or higher for once, n = 2636	49	2.35	84	4.00
Persistent trace or higher for two or more times, n = 952	20	2.67	60	7.94
p-value for log-rank test	<0.001		<0.001	

### Association analysis of proteinuria with MI or all-cause mortality

In the Cox regression analysis for persistent pattern of proteinuria, both occasional and persistent positive findings in proteinuria were shown to increase the risk of MI and all-cause mortality. Adjustment for covariates attenuated the effect size and made the association between persistent positive findings in proteinuria and MI lose statistical significance. At the same time, hazard ratio (HR) for persistent proteinuria was shown to be lower than that for occasional proteinuria. However, the associations between occasional positive findings in proteinuria and MI, as well as both occasional and persistent proteinuria and all-cause mortality, were still statistically significant. We can observe a dose-response relationship from occasional to persistent proteinuria for all-cause mortality (p-value for trend <0.001) (Table 3).

**Table 3:** Risks for myocardial infarction and all-cause mortality by persistent pattern of positive finding in proteinuria during follow-up.

Models^*^	Negative through follow-up	Occasional trace or higher for once [HR(95% CI)]	Persistent trace or higher for two or more times [HR(95% CI)]	p-value for trend
**Myocardial infarction**				
Model 1	Reference	1.71 (1.23, 2.36)	1.92 (1.21, 3.07)	<0.001
Model 2	Reference	1.62 (1.17, 2.25)	1.64 (1.02, 2.62)	0.002
Model 3	Reference	1.44 (1.03, 2.00)	1.20 (0.73, 1.96)	0.11
**All-cause mortality**				
Model 1	Reference	1.60 (1.25, 2.05)	3.20 (2.41, 4.23)	<0.001
Model 2	Reference	1.50 (1.17, 1.92)	2.58 (1.94, 3.43)	<0.001
Model 3	Reference	1.42 (1.10, 1.83)	2.23 (1.66, 3.01)	<0.001

*Note*: ^*^Model 1 was univariable association analysis; Model 2 was adjusted for age and gender; Model 3 was adjusted for variables in model 2 plus high school or above, current smoking, body mass index, mean blood pressure, use of blood pressure lowering agents, use of glucose lowering agents, fasting blood glucose, natural log-transformed triglyceride, low density lipoprotein cholesterol and estimated glomerular filtration rate levels at baseline examination.

Abbreviations: HR, Hazard ratio; CI, Confidence interval.

In regard to the degree of proteinuria, the same population was used as in the analysis for persistent pattern. The numbers of patients in the negative, trace or one plus or higher proteinuria group in the 2006–2007 health examination circle were 15,681 (94.62%), 789 (4.76%) and 103 (0.62%), respectively. The corresponding numbers were 10,835 (86.83%), 1,288 (10.32%) and 355 (2.85%) in the 2008–2009 circle, 13,194 (91.01%), 979 (6.75%) and 325 (2.24%) in the 2010–2011 circle, and 13,338 (92.62%), 644 (4.47%) and 419 (2.91%) in the 2012–1013 circle. A dose-response relationship was observed from trace to one plus or higher in proteinuria for both MI and all-cause mortality (p-values for trend < 0.05). However, the association between trace or one plus or higher in proteinuria and MI was not statistically significant after adjustment for covariates (Table 4).

**Table 4:** Risks for myocardial infarction and all-cause mortality by degree of proteinuria during follow-up.

Models^*^	Negative in urine dipstick test	Trace in urine dipstick test [HR(95% CI)]	One plus or higher in urine dipstick test [HR(95% CI)]	p-value for trend
**Myocardial infarction**				
Model 1	Reference	1.67 (1.06, 2.64)	2.76 (1.50, 5.07)	<0.001
Model 2	Reference	1.50 (0.95, 2.37)	2.41 (1.31, 4.45)	0.002
Model 3	Reference	1.26 (0.79, 2.00)	1.85 (0.99, 3.45)	0.04
**All-cause mortality**				
Model 1	Reference	2.50 (1.84, 3.40)	4.90 (3.58, 6.72)	<0.001
Model 2	Reference	1.94 (1.42, 2.65)	3.92 (2.86, 5.38)	<0.001
Model 3	Reference	1.80 (1.31, 2.48)	3.34 (2.40, 4.65)	<0.001

*Note*: ^*^Model 1 was univariable association analysis; Model 2 was adjusted for age and gender; Model 3 was adjusted for variables in model 2 plus high school or above, current smoking, body mass index, mean blood pressure, use of blood pressure lowering agents, use of glucose lowering agents, fasting blood glucose, natural log-transformed triglyceride, low density lipoprotein cholesterol and estimated glomerular filtration rate levels at baseline examination.

Abbreviations: HR, Hazard ratio; CI, Confidence interval.

### Association analysis of proteinuria with MI or all-cause mortality stratified by prediabetes or diabetes

We stratified the population by with pre-diabetes or diabetes. The associations between persistent pattern/degree of proteinuria and the adverse outcomes were more obvious among population with diabetes than those with pre-diabetes (Supplementary Tables S2–S5).

## Discussion

In this study, we evaluated the association of dipstick proteinuria with MI and all-cause mortality among a general population with diabetes or pre-diabetes. Proteinuria detected both occasionally and persistently was shown to increase the risk of MI and all-cause mortality. We also identified an association between elevated degree of proteinuria and the increased risk of the adverse outcomes.

Albumin is the main component of proteins in urine. Typically, urinary albumin excretion rate can be quantitatively measured by 24-hour urine albumin excretion or ACR, etc.^11^. However, process of the quantitative measurements can be complicated and costs are high. Urine dipstick test has the advantage of easy to conduct and un-expensive. Some studies have demonstrated a fairly good sensitivity and specificity of urine dipstick test compared to microalbuminuria reflected by ACR. Based on the AusDiab study, which included 10,944 Australian adults aged 25 years or older, White *et al*. systematically assessed the diagnostic accuracy of dipstick proteinuria. Using ACR ≥30 mg/g as the reference, the sensitivity of dipstick finding of trace or higher was 69.4%(95% CI: 65.9–72.7%) and the specificity was 86.8% (95% CI:86.1–87.4%). The corresponding sensitivity and specificity for 1+ or higher in dipstick test were 57.8% (95% CI: 54.1–61.4%) and 95.4% (95% CI: 95.0–95.8%), respectively. Meanwhile, the negative finding of dipstick test has a negative predictive value of 97.6% (95% CI: 97.2–97.9%) for ACR ≥30 mg/g^9^. Another study, which is based on the South Korean National Health and Nutrition Examination Survey, got a little lower sensitivity (43.6%) and higher specificity (93.6%) of dipstick finding of trace or higher with ACR ≥30 mg/g as the reference^12^.

Albuminuria/proteinuria is an established risk factor for all-cause and cardiovascular mortality among general population, which has been demonstrated by a comprehensive analysis involving 41 individual studies around the world. In the above mentioned study, consistent associations were recorded from studies with dipstick test or ACR^5^. With regard to the high risk population, van der Velde and colleagues reported the association between high albuminuria and all-cause or cardiovascular mortality among population with hypertension or diabetes in a meta-analysis^13^. Another meta-analysis, which focused only on the diabetic patients, also detected that microalbuminuria and macroalbuminuria can increase the risk of both cardiovascular and all-cause mortality^14^. When coronary heart disease was treated as the outcome, a meta-analysis including 169,949 individuals found that the presence of proteinuria is significantly and continuously associated with the increased risk of the disease^15^. Several other individual studies also reported that increased ACR at baseline, sustained microalbuminuria over time or microalbuminuria progressed to macroalbuminuria were associated with increased risk of cardiovascular events among diabetic patients^16–18^. The results of our study were largely consistent with the above mentioned studies and provided additional evidence about proteinuria in a longitudinal pattern. The exception is the non-significant association between persistent proteinuria and MI. In a comprehensive analysis of the association between proteinuria and myocardial infarction, Brenda and colleagues demonstrated that risk of MI consistently increased through elevated proteinuria levels (reflected by dipstick test or ACR) and the association was independent of eGFR levels^10^. We assume that the null association in our study is due to the limited number of events, as there are only 20 MI identified in the group with persistent proteinuria. With these limited number of events, we observed a significant association among people with diabetes in the stratified analysis, but not among pre-diabetes and total population.

The Steno hypothesis suggests that albuminuria reflects wide-spread vascular damage^19^. A number of studies have demonstrated that markers of endothelial dysfunction, systemic inflammation, thrombogenesis and oxidative stress were correlated with albuminuria/proteinuria^20,21^. Some recent studies proposed that degradation of endothelial glycocalyx served as the key mechanism linking albuminuria with cardiovascular diseases^22^. Endothelial glycocalyx is a layer of polysaccharide gel, which attaches to the vascular endothelial luminal surface and acts as a barrier against albumin filtration. In the condition of metabolic disorders, such as diabetes mellitus and hypertension, endothelial cells are activated, leading to dysfunction of glycocalyx through the increased activity of enzymes. In kidneys, loss of glycocalyx makes more protein enter the subpodocyte space and leads to protein uptake by podocytes, which induces apoptosis of podocytes and proliferation of parietal epithelial cells. It is believed that albuminuria aroused by degradation of glycocalyx is an important cause of kidney disease progression, featured by podocytes and tubulointerstitial damage, rather than just a marker of kidney disease progression. To the extent of all capillary beds, loss of glycocalyx leads to increased transport of albumin and filtration of lipoproteins into the subendothelial space^22^. An example of the process is the vascular lipoprotein deposition and atherosclerosis in internal carotid arteries^23^. Several studies have determined the association between albuminuria and structural change and dysfunction of the heart. Among a population with normal left ventricular ejection fraction and wall motion, increased level of ACR was found to be associated with adverse cardiac mechanics^24^. In another study including patients with heart failure but with preserved ejection fraction, albuminuria is associated with cardiac remodeling and left and right ventricular dysfunction^25^. In our study, MI, a major kind of CVD, was used to reflect the cardiovascular damage. Hence, our study findings were consistent with the pathophysiological mechanisms of the effect of albuminuria.

Along with the continuous increase in prevalence of hypertension and diabetes in recent years in China^26^, the spectrum of CKD of the country was supposed to transit towards a metabolic disease dominant situation. A recent study provided evidence supporting the assumption by systematically analyzing ICD code of 35.3 million hospitalized patients from a nation-wide registry of China from 2009 through 2015. The study found that the number of CKD related to DM surpassed that of CKD related to glomerulonephritis in 2010 and the gap continued to extend through the end of the study^27^. As the spectrum of CKD is approaching the situation in developed countries, it may have great public health importance to make early recognition of CKD to improve the awareness and ultimately to guide proper health interventions. An US based cost-effectiveness study recommended screening for proteinuria followed by proper use of medications among populations with older age, hypertension or diabetes^28^. We assume that dipstick test may be a reasonable way to be used in high risk populations in the setting of primary health care services, especially among low and middle income countries.

Although our study has some advantages, e.g. having a large sample size and a longitudinal design to obtain time-updated proteinuria, there are some limitations that should be admitted. First, our study was based on a population with a majority of men and that may limit generalizability of the study findings to women. Second, the presence of proteinuria was not confirmed in repeated measurements during a short time period, e.g. 3–6 months, thus it will be subject to some uncertainties for the exposure status due to a day-to-day variability in urinary albumin excretion. Third, people with impaired glucose tolerance, while with a normal fasting glucose level, would have been missed in the current analysis. That would cause a selection bias of the population. Fourth, we failed to record stroke as the outcome of our study, which may limit our study generalizability regarding this major type of CVD.

In conclusion, our study found that proteinuria detected by urine dipstick test is associated with increased risk of MI and all-cause mortality among a general Chinese population with pre-diabetes or diabetes. The results added further evidence regarding prognosis of CKD among the high risk population and provided supportive evidence for conducting mass-screening by the cost-effective way of identifying kidney damage among general populations.

## Methods

### Study design and population

**Figure 1:**
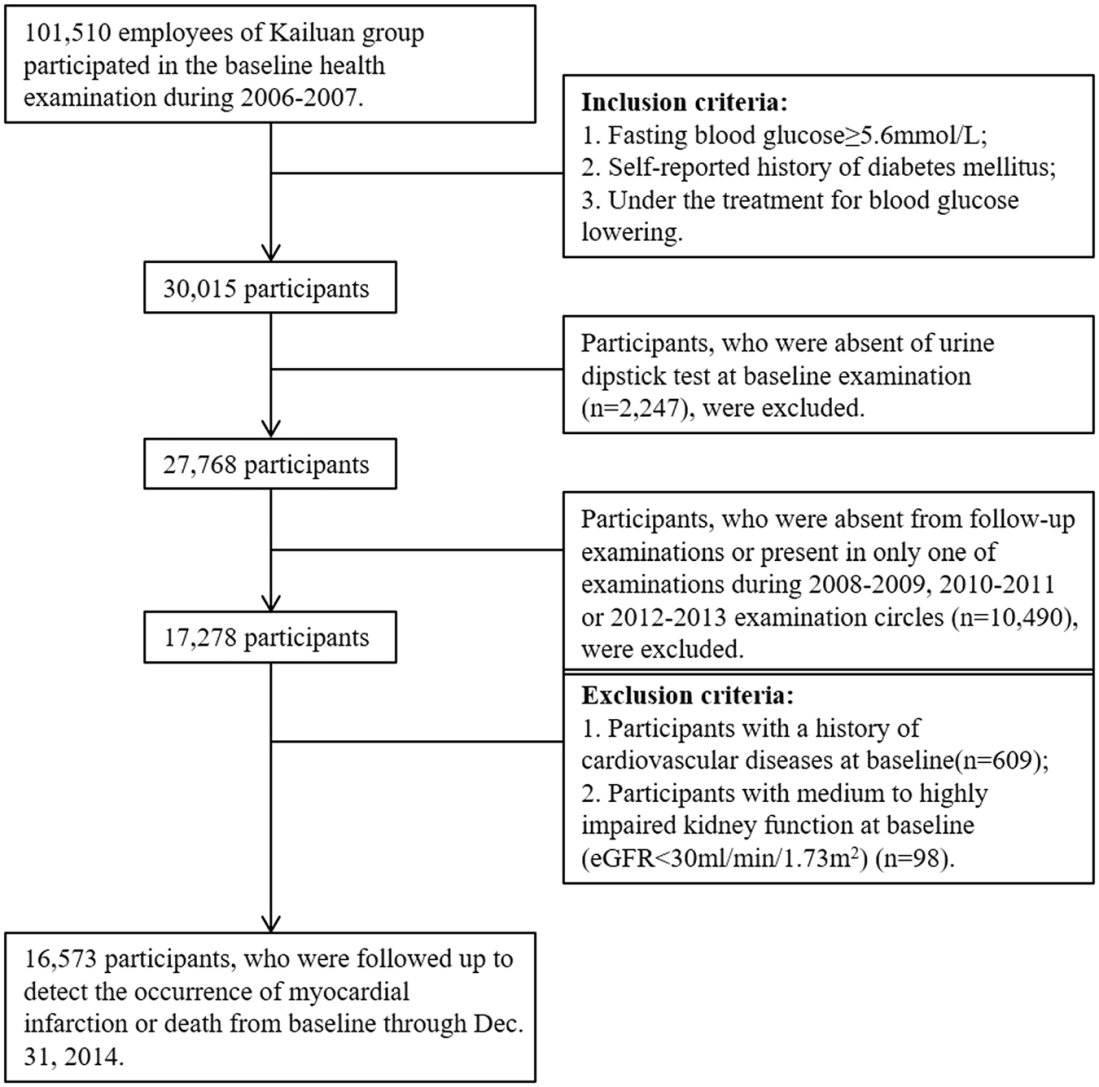
Flow chart of the study participants.

Our study is based on the Kailuan study, which is a prospective cohort study based on the Kailuan population in Tangshan, a large industrial city located in Hebei province of China. The detailed study design and characteristics of the study population have been described previously^29^. Briefly, 101,510 employees aged above 18 years (including the retired) in Kailuan group participated in the health examination and built their health records from June 2006 through October 2007 (baseline of the current study). Items of examination included face to face questionnaire investigation, clinical examination and laboratory tests, which was conducted in Kailuan General Hospital and its 10 affiliated hospitals. All the participants underwent health examinations biennially until December 31, 2013. First, we included people with abnormal FBG (≥5.6 mmol/L), and/or self-reported history of diabetes mellitus and/or currently under blood glucose lowering therapy (n = 30,015), as well as with the availability of data on urine dipstick test (n = 27,768) based on baseline examination circle. Second, we included people, who participated in at least two follow-up examinations of the 2008–2009, 2010–2011 and 2012–2013 examination circles (n = 17,278). Third, we excluded people with a history of CVD (n = 609). As medium to highly impaired kidney function could pose a high risk of CVD and these population accounted for only a small proportion in the current study, we also excluded individuals with a baseline eGFR of <30 ml/min/1.73 m^2^ (n = 98). The detailed procedure of participant selection was shown in Fig. 1. Totally, 16,573 participants were included in the final analysis. The study was conducted according to the guidelines of Helsinki declaration and was approved by the ethics committee of Kailuan General Hospital and Peking University First Hospital. The informed content was obtained from each participant before the health examination.

### Determination of proteinuria and covariates

A face to face interview was administered by the trained doctors or nurses by using standard questionnaire with information of demographic characteristics, lifestyles and disease histories. Height and weight were measured and BMI was calculated as body weight (kg) divided by height in square meters (m^2^). Blood pressure was measured using a standardized mercury sphygmomanometer according to the standard procedures. SBP and DBP were taken at a 5-minute interval for two times after participants sitting for at least 5 minutes. The average of the two readings was used for analysis. If the two measurements differed by >5 mmHg, then the third measurement was conducted and the average of the three readings was used. Mean blood pressure (MBP) was calculated as one third of SBP plus two thirds of DBP. A single random mid-stream morning urine sample was collected from each participant. Urine protein concentration was assessed by dry chemistry method with the test assay of H12-MA (Changchun Dirui Medical Technology Co., Ltd. Changchun, China). The lower detecting limit is 15 mg/dL. All the urine samples were measured using a urine analyzer (N-600, Dirui, Changchun, China) at the central laboratory of the Kailuan hospital. The levels of the semi-quantitative dipstick test were recorded as negative (<15 mg/dL), trace (15–29 mg/dL), 1+ (30–300 mg/dL), 2+ (300–1000 mg/dL), or 3+ (>1000 mg/dL). A positive result was defined as trace or higher in the analysis for persistent pattern of proteinuria. Blood samples were collected from the antecubital vein after an at least 8 hours fast. FBG, TG, LDL-C and serum creatinine were tested using a Hitachi 7600 auto-analyzer (Hitachi; Tokyo, Japan) at the central laboratory of the Kailuan General Hospital. eGFR was estimated by using the two race Chronic Kidney Disease Epidemiology Equation^30^.

CKD stages were categorized by the level of eGFR as follows, stage 1 as eGFR ≥90 ml/min/1.73 m^2^, stage 2 as eGFR between 60 and 90 ml/min/1.73 m^2^, stage 3 as eGFR between 30 and 60 ml/min/1.73 m^2^.

### Assessment of outcomes

Adverse outcomes of non-fatal MI and all-cause mortality were recorded. Information on the MI event was collected from biennial personal interviews, discharge summaries from the 11 hospitals, and medical records from medical insurance. The definition of MI was applied according to the standard criteria^31^. The criteria were consistently applied across all the 11 hospitals. Death information were collected from vital statistics offices in local government. All outcomes were validated by the Data Safety Monitoring Board and the Arbitration Committee for Clinical Outcomes. We followed the participants from the date of the baseline health examination through December 31, 2014, or to the events of interest.

### Statistical analysis

The population was stratified by the persistence of positive findings in proteinuria during follow-up as no proteinuria, occasional proteinuria for once, persistent proteinuria for two or more times. Continuous variables are presented as mean (SD) and compared among groups by using ANOVA test. If the variable does not satisfy the normal distribution, the median (interquartile range) and Kruskal-Wallis rank sum test will be used. Categorical variables were presented as frequency (percent) and compared by using *χ* ^2^ tests. The incidence rate of the adverse outcomes was calculated and compared among groups by using the log-rank test. Cox proportional hazards regression model was used to estimate the association between persistence or degree of proteinuria and the adverse outcomes. In order to reflect the changing status of proteinuria, a time-dependent variable was used for degree of proteinuria, i.e. proteinuria measurements from each circle of health examination were used. In regard to the endpoint of MI, the Fine and Gray model was used to treat death as a competing risk, which impeded the occurrence of MI^32^. Proportional hazards assumptions of the Cox regression model were verified by testing the interaction with time using the likelihood ratio test, which yielded non-significant p-values. HRs and 95% CI were reported. We included covariates investigated at baseline into the Cox regression model step by step. Model 1 was the univariable mode l. Model 2 included age (continuous) and sex (male versus female). Model 3 included covariates in model 2 plus education (high school or above versus below high school level), current smoking (yes versus no), current use of blood pressure lowering agents (yes versus no), current use of glucose lowering agents (yes versus no), BMI (continuous), MBP (continuous), FBG (continuous), natural log-transformed TG (continuous), LDL-C (continuous) and CKD stages (stage 3, stage 2 versus stage 1). All p-values were two-sided, and p-value of statistical significance was 0.05. All statistical analyses were performed by using SAS 9.4 (SAS Institute; Cary, NC).

### Data availability

The datasets generated during and/or analysed during the current study are not publicly available due to agreement between the study collaboration centers but are available from the corresponding author on reasonable request.

## Electronic supplementary material

Supplementary tables
