# Supplementary material for: Dipstick proteinuria and risk of myocardial infarction and all-cause mortality in diabetes or pre-diabetes: a population-based cohort study
Source: Sci Rep. 2017 Sep 20;7:11986. doi: 10.1038/s41598-017-12057-4 (PMC5607308; doi:10.1038/s41598-017-12057-4)
Supplement: Supplementary file 1 — Supplementary tables [file 41598_2017_12057_MOESM1_ESM.pdf]

Dipstick proteinuria and risk of myocardial infarction and all-cause mortality in diabetes or pre-diabetes: a population-based cohort study

Jinwei Wang, PhD[1†], Junjuan Li, MD[2†], Anxin Wang, PhD[3, 4], Jianli Wang, MD[2], Yaozheng Yang, MD[1], Shuohua Chen, MD[5], Shouling Wu, MD[5], Minghui Zhao, MD, PhD[1], Xiuhua Guo, PhD[3], Luxia Zhang, MD, MPH[1*]

1. Renal Division, Department of Medicine, Peking University First Hospital; Institute of Nephrology, Peking University; Key Laboratory of Renal Disease, National Health and Family Planning Commission of the People's Republic of China; Key Laboratory of Chronic Kidney Disease Prevention and Treatment, Ministry of Education, Beijing 100034, China
2. Department of Nephrology, Kailuan General Hospital Affiliated to North China University of science and technology, Tangshan 063000, China
3. Department of Epidemiology and Health Statistics, School of Public Health, Capital Medical University; Municipal Key Laboratory of Clinical Epidemiology, Beijing 100069, China
4. Department of Neurology, Beijing Tiantan Hospital, Capital Medical University, Beijing 100050, China
5. Department of Cardiology, Kailuan General Hospital Affiliated to North China University of science and technology, Tangshan 063000, China

[†]These authors contributed equally to this work.

[*]Correspondence:

Luxia Zhang, MD, MPH

Renal Division, Department of Medicine, Peking University First Hospital;

8 Xishiku Street, Xicheng District, Beijing 100034, China;

Phone: +86-10-83575817;

Fax: +86-10-66551055;

Email: zhanglx@bjmu.edu.cn

Supplementary table S1. Characteristics of participants excluded from or included in the study

Characteristics	Participants excluded from the study, n=13442	Participants included in the study, n=16573	p-value
Age(years)	56.98(11.72)	51.16(10.63)	<0.001
Male	11816(87.9%)	13629(82.24%)	<0.001
High school and above	1885(14.52%)	3222(20.01%)	<0.001
Current smoking	4714(35.95%)	5884(36.24%)	0.60
BMI(kg/m^2)	25.65(3.51)	25.87(3.38)	<0.001
SBP(mmHg)	138.39(22.50)	133.82(20.54)	<0.001
DBP(mmHg)	86.21(12.30)	85.13(11.62)	<0.001
FBG(mmol/L)	7.13(2.30)	6.96(2.07)	<0.001
Current use of blood pressure lowering agents	2583(20.36%)	2029(12.91%)	<0.001
Current use of glucose lowering agents	1289(9.59%)	1191(7.19%)	<0.001
TG(mmol/L)	1.44(1.01,2.2)	1.48(1.03,2.3)	<0.001
LDL-C(mmol/L)	2.58(0.94)	2.44(0.87)	<0.001
Serum creatinine(mg/dl)	1.08(0.52)	1.01(0.25)	<0.001
eGFR(ml/min/1.73m^2)	78.57(64.27,94.13)	82.26(68.68,98.14)	<0.001
eGFR categories			<0.001
≥90ml/min/1.73m^2	4262(31.79%)	6210(37.52%)	
60-90ml/min/1.73m^2	6641(49.54%)	8431(50.95%)	
<60ml/min/1.73m^2	2269(16.93%)	1908(11.53%)	

Note: Data were presented as mean(standard deviation) or median(interquartile range) for continuous variables and frequency(proportion) for categorical variables. Abbreviations: BMI=Body mass index, SBP=systolic blood pressure, DBP=diastolic blood pressure, FBG=Fasting blood glucose, TG=Triglyceride, LDL-C= Low density lipoprotein cholesterol, eGFR=estimated Glomerular filtration rate.

Supplementary table S2. Risks for myocardial infarction and all-cause mortality by persistent pattern of positive finding in proteinuria during follow-up among participants with pre-diabetes

Models*	Negative through follow-up	Occasional trace or higher for once[HR(95%CI)]	Persistent trace or higher for two or more times[HR(95%CI)]	p-value for trend
Myocardial infarction				
Model1	Reference	1.79(1.14,2.80)	1.13(0.41,3.08)	0.05
Model2	Reference	1.76(1.12,2.78)	0.98(0.35,2.69)	0.10
Model3	Reference	1.49(0.94,2.36)	0.74(0.27,2.05)	0.52
All-cause mortality				
Model1	Reference	1.65(1.16,2.35)	3.12(1.93,5.03)	<0.001
Model2	Reference	1.61(1.13,2.29)	2.63(1.63,4.24)	<0.001
Model3	Reference	1.59(1.11,2.28)	2.55(1.59,4.11)	<0.001

Note: *Model1 was univariable association analysis; Model2 was adjusted for age and gender; Model3 was adjusted for variables in model2 plus high school and above, current smoking, body mass index, mean blood pressure, use of blood pressure lowering agents, fasting blood glucose, natural log-transformed triglyceride, low density lipoprotein cholesterol and estimated glomerular filtration rate levels at baseline examination.

Abbreviations: HR, Hazard ratio; CI, Confidence interval.

Supplementary table S3. Risks for myocardial infarction and all-cause mortality by degree of proteinuria during follow-up among participants with pre-diabetes

Models*	Negative in urine dipstick test	Trace in urine dipstick test[HR(95%CI)]	One plus or higher in urine dipstick test[HR(95%CI)]	p-value for trend
Myocardial infarction				
Model1	Reference	1.23(0.57,2.67)	0.71(0.10,5.14)	0.89
Model2	Reference	1.14(0.53,2.47)	0.61(0.08,4.46)	0.90
Model3	Reference	0.96(0.44,2.09)	0.45(0.06,3.29)	0.49
All-cause mortality				
Model1	Reference	2.39(1.47,3.90)	5.93(3.57,9.85)	<0.001
Model2	Reference	2.01(1.23,3.29)	4.96(3.00,8.19)	<0.001
Model3	Reference	1.96(1.19,3.23)	4.87(2.94,8.08)	<0.001

Note: *Model1 was univariable association analysis; Model2 was adjusted for age and gender; Model3 was adjusted for variables in model2 plus high school and above, current smoking, body mass index, mean blood pressure, use of blood pressure lowering agents, fasting blood glucose, natural log-transformed triglyceride, low density lipoprotein cholesterol and estimated glomerular filtration rate levels at baseline examination.

Abbreviations: HR, Hazard ratio; CI, Confidence interval.

Supplementary table S4. Risks for myocardial infarction and all-cause mortality by persistent pattern of positive finding in proteinuria during follow-up among participants with diabetes

Models*	Negative through follow-up	Occasional trace or higher for once[HR(95%CI)]	Persistent trace or higher for two or more times[HR(95%CI)]	p-value for trend
Myocardial infarction				
Model1	Reference	1.45(0.91,2.31)	1.76(1.02,3.05)	0.02
Model2	Reference	1.38(0.87,2.21)	1.63(0.94,2.82)	0.04
Model3	Reference	1.29(0.81,2.07)	1.46(0.82,2.62)	0.14
All-cause mortality				
Model1	Reference	1.36(0.97,1.92)	2.37(1.66,3.37)	<0.001
Model2	Reference	1.30(0.92,1.83)	2.18(1.52,3.11)	<0.001
Model3	Reference	1.25(0.88,1.77)	2.08(1.43,3.03)	<0.001

Note: *Model1 was univariable association analysis; Model2 was adjusted for age and gender; Model3 was adjusted for variables in model2 plus high school and above, current smoking, body mass index, mean blood pressure, use of blood pressure lowering agents, use of glucose lowering agents, fasting blood glucose, natural log-transformed triglyceride, low density lipoprotein cholesterol and estimated glomerular filtration rate levels at baseline examination.
Abbreviations: HR, Hazard ratio; CI, Confidence interval.

Supplementary table S5. Risks for myocardial infarction and all-cause mortality by degree of proteinuria during follow-up among participants with diabetes

Models*	Negative in urine dipstick test	Trace in urine dipstick test[HR(95%CI)]	One plus or higher in urine dipstick test[HR(95%CI)]	p-value for trend
Myocardial infarction				
Model1	Reference	1.75(0.99,3.10)	3.05(1.58,5.86)	<0.001
Model2	Reference	1.63(0.92,2.90)	2.91(1.51,5.60)	<0.001
Model3	Reference	1.52(0.85,2.72)	2.70(1.37,5.32)	0.003
All-cause mortality				
Model1	Reference	2.08(1.40,3.09)	3.26(2.18,4.88)	<0.001
Model2	Reference	1.69(1.13,2.52)	2.97(1.97,4.47)	<0.001
Model3	Reference	1.68(1.11,2.53)	2.77(1.82,4.19)	<0.001

Note: *Model1 was univariable association analysis; Model2 was adjusted for age and gender; Model3 was adjusted for variables in model2 plus high school and above, current smoking, body mass index, mean blood pressure, use of blood pressure lowering agents, use of glucose lowering agents, fasting blood glucose, natural log-transformed triglyceride, low density lipoprotein cholesterol and estimated glomerular filtration rate levels at baseline examination.

Abbreviations: HR, Hazard ratio; CI, Confidence interval.
